# (*S*)-2-(1-Hydroxy­ethyl)benzimid­azolium dihydrogen phosphate

**DOI:** 10.1107/S1600536809028451

**Published:** 2009-07-29

**Authors:** Rong Xia

**Affiliations:** a Ordered Matter Science Research Center, College of Chemistry and Chemical Engineering, Southeast University, Nanjing 210096, People’s Republic of China

## Abstract

The asymmetric unit of the title compound, C_9_H_11_N_2_O^+^·H_2_PO_4_^−^, is built up from a 2-(1-hydroxy­ethyl)benz­imid­a­zol­ium cation and a dihydrogen phosphate anion which are connected by an N—H⋯O hydrogen bond. The cation is roughly planar, the dihedral angle between the rings being only 1.4 (2)°. The *S* configuration is deduced from the synthetic pathway and supported by the refinement of the Flack parameter. Inter­molecular O—H⋯O and N—H⋯O hydrogen bonds build up a three-dimensionnal network.

## Related literature

For the biological and pharmaceutical activity of imidazole and benzimidazole derivatives, see: Rodembusch *et al.* (2004 ▶); Gong *et al.* (2005 ▶); Chen (2005 ▶); Belmar *et al.* (1999 ▶). For the synthesis and crystal structure of (±)-1-(1*H*-benzimidazol-2-yl)ethanol, see: Xia & Xu (2008 ▶).
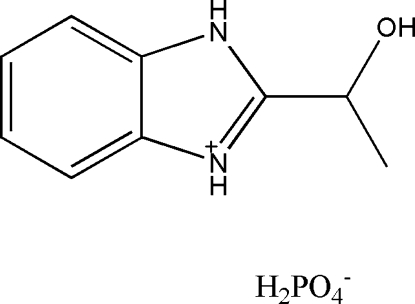


## Experimental

### 

#### Crystal data

C_9_H_11_N_2_O^+^·H_2_PO_4_^−^
*M* *_r_* = 260.18
Orthorhombic, 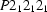

*a* = 4.5869 (13) Å
*b* = 15.749 (5) Å
*c* = 15.876 (5) Å
*V* = 1146.8 (6) Å^3^
*Z* = 4
Mo *K*α radiation
μ = 0.25 mm^−1^
*T* = 293 K
0.20 × 0.20 × 0.20 mm

#### Data collection

Rigaku SCXmini diffractometer
Absorption correction: multi-scan (*CrystalClear*; Rigaku, 2005 ▶) *T*_min_ = 0.951, *T*_max_ = 0.953
11500 measured reflections
2565 independent reflections
1749 reflections with *I* > 2σ(*I*)
*R*_int_ = 0.125

#### Refinement

*R*[*F* ^2^ > 2σ(*F* ^2^)] = 0.052
*wR*(*F* ^2^) = 0.118
*S* = 0.82
2565 reflections
159 parameters
H-atom parameters constrained
Δρ_max_ = 0.39 e Å^−3^
Δρ_min_ = −0.37 e Å^−3^
Absolute structure: Flack (1983 ▶), 1030 Friedel pairs
Flack parameter: 0.16 (17)

### 

Data collection: *CrystalClear* (Rigaku, 2005 ▶); cell refinement: *CrystalClear*; data reduction: *CrystalClear*; program(s) used to solve structure: *SHELXS97* (Sheldrick, 2008 ▶); program(s) used to refine structure: *SHELXL97* (Sheldrick, 2008 ▶); molecular graphics: *SHELXTL* (Sheldrick, 2008 ▶); software used to prepare material for publication: *SHELXTL*.

## Supplementary Material

Crystal structure: contains datablocks I, global. DOI: 10.1107/S1600536809028451/dn2468sup1.cif

Structure factors: contains datablocks I. DOI: 10.1107/S1600536809028451/dn2468Isup2.hkl

Additional supplementary materials:  crystallographic information; 3D view; checkCIF report

## Figures and Tables

**Table 1:** Hydrogen-bond geometry (Å, °)

*D*—H⋯*A*	*D*—H	H⋯*A*	*D*⋯*A*	*D*—H⋯*A*
O3—H3⋯O4^i^	0.82	1.76	2.532 (4)	156
O2—H2⋯O5^ii^	0.82	1.76	2.539 (3)	157
N2—H2*A*⋯O4	0.86	1.88	2.729 (4)	167
N1—H1⋯O5^iii^	0.86	1.81	2.659 (4)	167
O1—H1*A*⋯O2^iv^	0.82	2.23	2.971 (4)	150

Symmetry codes: (i) 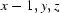; (ii) 
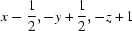
; (iii) 
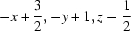
; (iv) 
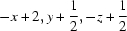
.

**Fig. 1.**
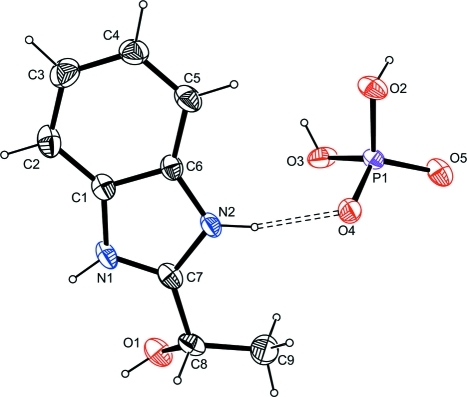
Molecular structure of the title compound with the atom labeling scheme. Displacement ellipsoids are drawn at the 30% probability level. H atoms are represented as small spheres of arbitrary radii. H bond is shown as dashed line.

**Fig. 2.**
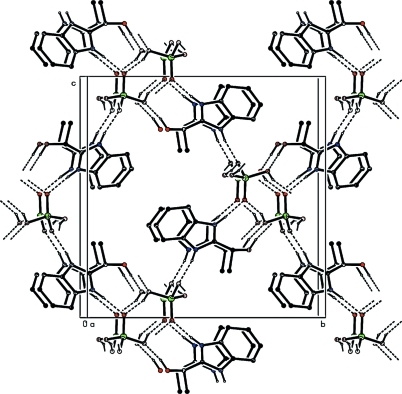
Packing diagram of the title compound viewed along the a axis. Intermolecular O—H···O hydrogen bonds and N—H···O hydrogen bonds are shown as dashed lines. H atoms not involved in hydrogen bondings have been omitted for clarity.

## supplementary crystallographic information

### Comment

The benzimidazoles, benzothiazoles, and benzoxazoles can be utilized as not only a wide variety of biologically active and medicinally significant compounds but also as advanced materials including non-linear optics (NLO), organic light-emitting diodes (OLED), and liquid crystals (Rodembusch *et al.*, 2004; Gong *et al.*, 2005; Chen, 2005; Belmar *et al.*, 1999).

The title compound is built up from a dihydrogen phosphate anion and a (1H-benzimidazol-2-yl)ethanolium cation which are connected by a N-H···O hydrogen bond (Fig. 1).The S absolute configuration is deduced from the synthetic pathway and supported by the refinement of the Flack parameter (Flack, 1983). The phenyl ring and imidazole ring are roughly planar, making a dihedral angle of only1.4°. All bond lengths and angels are normal.

The molecules are connected *via* O—H···O and N—H···O hydrogen bonds making a three dimensionnal network (Table 1, Fig. 2).

### Experimental

A solution of phosphoric acid (1 mmol) in water was added to a methanol solution of *L*-(-)-1-(1*H*-Benzimidazol-2-yl)ethanol (1 mmol), and then the mixture was stirred for half an hour at room temperature. The mixture was then filtered and the filtrate was evaporated at room temperature for a period of one month.Crystals suitable for X-ray diffraction analysis were obtained then. *L*-(-)-1-(1*H*-Benzimidazol-2-yl)ethanol was synthesized by the reaction of Benzene-1, 2-diamine and Ethyl *L*-(-)-lactate(*R*. Xia, *et al.*, 2008).

### Refinement

All H atoms attached to C and O atom were fixed geometrically and treated as riding with C—H = 0.93–0.98 Å, O—H = 0.82 Å and with *U*_iso_(H) = 1.2Ueq(C) or 1.5Ueq(C, O) for methyl and hydroxyl.

The su on the Flack parameter is rather high, however the value of the parameter agrees with the S configuration. Moreover, inverting the configuration leads to the value 0.88 close to 1.

### Crystal data

**Table:**

C_9_H_11_N_2_O^+^·H_2_PO_4_^−^	*F*(000) = 544
*M**_r_* = 260.18	*D*_x_ = 1.507 Mg m^−^^3^
Orthorhombic, *P*2_1_2_1_2_1_	Mo *K*α radiation, λ = 0.71073 Å
Hall symbol: P 2ac 2ab	Cell parameters from 3144 reflections
*a* = 4.5869 (13) Å	θ = 2.6–27.4°
*b* = 15.749 (5) Å	µ = 0.25 mm^−^^1^
*c* = 15.876 (5) Å	*T* = 293 K
*V* = 1146.8 (6) Å^3^	Block, pale yellow
*Z* = 4	0.20 × 0.20 × 0.20 mm

### Data collection

**Table:**

Rigaku SCXmini diffractometer	2565 independent reflections
Radiation source: fine-focus sealed tube	1749 reflections with *I* > 2σ(*I*)
graphite	*R*_int_ = 0.125
Detector resolution: 13.6612 pixels mm^-1^	θ_max_ = 27.3°, θ_min_ = 2.6°
ω scans	*h* = −5→5
Absorption correction: multi-scan (*CrystalClear*; Rigaku, 2005)	*k* = −20→20
*T*_min_ = 0.951, *T*_max_ = 0.953	*l* = −20→20
11500 measured reflections	

### Refinement

**Table:**

Refinement on *F*^2^	Secondary atom site location: difference Fourier map
Least-squares matrix: full	Hydrogen site location: inferred from neighbouring sites
*R*[*F*^2^ > 2σ(*F*^2^)] = 0.052	H-atom parameters constrained
*wR*(*F*^2^) = 0.118	*w* = 1/[σ^2^(*F*_o_^2^) + (0.0314*P*)^2^] where *P* = (*F*_o_^2^ + 2*F*_c_^2^)/3
*S* = 0.82	(Δ/σ)_max_ < 0.001
2565 reflections	Δρ_max_ = 0.39 e Å^−^^3^
159 parameters	Δρ_min_ = −0.37 e Å^−^^3^
0 restraints	Absolute structure: Flack (1983), 1030 Friedel pairs
Primary atom site location: structure-invariant direct methods	Flack parameter: 0.16 (17)

### Special details

**Table:**

Geometry. All esds (except the esd in the dihedral angle between two l.s. planes) are estimated using the full covariance matrix. The cell esds are taken into account individually in the estimation of esds in distances, angles and torsion angles; correlations between esds in cell parameters are only used when they are defined by crystal symmetry. An approximate (isotropic) treatment of cell esds is used for estimating esds involving l.s. planes.
Refinement. Refinement of *F*^2^ against ALL reflections. The weighted *R*-factor *wR* and goodness of fit *S* are based on *F*^2^, conventional *R*-factors *R* are based on *F*, with *F* set to zero for negative *F*^2^. The threshold expression of *F*^2^ > σ(*F*^2^) is used only for calculating *R*-factors(gt) *etc*. and is not relevant to the choice of reflections for refinement. *R*-factors based on *F*^2^ are statistically about twice as large as those based on *F*, and *R*- factors based on ALL data will be even larger.

### Fractional atomic coordinates and isotropic or equivalent isotropic displacement parameters (Å^2^)

**Table:**

	*x*	*y*	*z*	*U*_iso_*/*U*_eq_	
P1	0.8027 (2)	0.34270 (6)	0.42082 (6)	0.0274 (2)	
O5	0.8944 (6)	0.34283 (14)	0.51179 (14)	0.0379 (7)	
O4	1.0419 (5)	0.36640 (16)	0.35969 (15)	0.0389 (7)	
O3	0.5477 (6)	0.40686 (15)	0.4094 (2)	0.0470 (8)	
H3	0.3968	0.3809	0.3992	0.070*	
O2	0.6959 (7)	0.25204 (15)	0.39269 (13)	0.0415 (7)	
H2	0.5776	0.2339	0.4270	0.062*	
N2	0.8351 (7)	0.44478 (17)	0.21853 (17)	0.0336 (7)	
H2A	0.9180	0.4266	0.2636	0.040*	
N1	0.7140 (7)	0.52823 (18)	0.11523 (16)	0.0369 (8)	
H1	0.7060	0.5722	0.0832	0.044*	
C8	1.0565 (9)	0.5932 (2)	0.2193 (2)	0.0385 (9)	
H8	1.2233	0.6030	0.1820	0.046*	
O1	0.8750 (6)	0.66679 (16)	0.21909 (17)	0.0514 (8)	
H1A	0.9367	0.7010	0.1844	0.077*	
C1	0.5597 (9)	0.4529 (2)	0.1022 (2)	0.0336 (9)	
C5	0.5154 (9)	0.3179 (2)	0.1774 (2)	0.0421 (10)	
H5	0.5643	0.2822	0.2219	0.051*	
C2	0.3599 (10)	0.4283 (3)	0.0413 (2)	0.0449 (11)	
H2B	0.3066	0.4640	−0.0027	0.054*	
C6	0.6383 (9)	0.3991 (2)	0.1692 (2)	0.0322 (9)	
C7	0.8737 (8)	0.5219 (2)	0.1839 (2)	0.0329 (9)	
C4	0.3198 (10)	0.2939 (2)	0.1167 (2)	0.0478 (11)	
H4	0.2345	0.2404	0.1200	0.057*	
C9	1.1665 (13)	0.5756 (2)	0.3069 (3)	0.0635 (14)	
H9A	1.2756	0.6237	0.3267	0.095*	
H9B	1.2900	0.5264	0.3060	0.095*	
H9C	1.0043	0.5656	0.3437	0.095*	
C3	0.2448 (10)	0.3478 (3)	0.0497 (2)	0.0523 (12)	
H3A	0.1127	0.3285	0.0096	0.063*	

### Atomic displacement parameters (Å^2^)

**Table:**

	*U*^11^	*U*^22^	*U*^33^	*U*^12^	*U*^13^	*U*^23^
P1	0.0291 (5)	0.0195 (4)	0.0336 (4)	−0.0002 (4)	−0.0001 (4)	0.0016 (4)
O5	0.0578 (18)	0.0220 (12)	0.0341 (13)	0.0091 (14)	0.0013 (13)	−0.0032 (10)
O4	0.0261 (14)	0.0505 (17)	0.0400 (15)	−0.0040 (13)	−0.0005 (12)	0.0139 (12)
O3	0.0274 (15)	0.0281 (14)	0.085 (2)	0.0010 (13)	−0.0108 (17)	−0.0035 (14)
O2	0.065 (2)	0.0229 (13)	0.0362 (14)	−0.0107 (15)	0.0061 (14)	−0.0083 (10)
N2	0.043 (2)	0.0280 (16)	0.0300 (15)	0.0026 (15)	−0.0003 (16)	0.0105 (12)
N1	0.053 (2)	0.0261 (16)	0.0315 (15)	0.0063 (17)	0.0011 (16)	0.0088 (12)
C8	0.038 (2)	0.0279 (19)	0.050 (2)	−0.0014 (19)	0.004 (2)	0.0047 (17)
O1	0.067 (2)	0.0256 (15)	0.0619 (19)	0.0066 (16)	0.0083 (17)	0.0083 (12)
C1	0.040 (2)	0.030 (2)	0.030 (2)	0.0079 (19)	0.0019 (18)	0.0006 (14)
C5	0.054 (3)	0.027 (2)	0.045 (2)	0.003 (2)	0.003 (2)	0.0048 (16)
C2	0.057 (3)	0.048 (2)	0.030 (2)	0.012 (2)	−0.003 (2)	0.0021 (17)
C6	0.036 (2)	0.0259 (18)	0.035 (2)	0.0062 (17)	0.0018 (17)	0.0025 (14)
C7	0.039 (2)	0.0258 (18)	0.0341 (19)	0.0044 (17)	0.0068 (17)	0.0041 (14)
C4	0.059 (3)	0.032 (2)	0.053 (3)	−0.008 (2)	0.001 (2)	−0.0043 (17)
C9	0.082 (4)	0.037 (2)	0.071 (3)	−0.010 (3)	−0.033 (3)	0.007 (2)
C3	0.057 (3)	0.050 (3)	0.050 (2)	−0.004 (3)	−0.012 (2)	−0.012 (2)

### Geometric parameters (Å, °)

**Table:**

P1—O5	1.504 (2)	C8—H8	0.9800
P1—O4	1.512 (3)	O1—H1A	0.8200
P1—O3	1.556 (3)	C1—C2	1.388 (5)
P1—O2	1.574 (2)	C1—C6	1.406 (4)
O3—H3	0.8200	C5—C4	1.370 (5)
O2—H2	0.8200	C5—C6	1.404 (5)
N2—C7	1.345 (4)	C5—H5	0.9300
N2—C6	1.395 (4)	C2—C3	1.380 (5)
N2—H2A	0.8600	C2—H2B	0.9300
N1—C7	1.317 (4)	C4—C3	1.403 (5)
N1—C1	1.397 (4)	C4—H4	0.9300
N1—H1	0.8600	C9—H9A	0.9600
C8—O1	1.427 (4)	C9—H9B	0.9600
C8—C9	1.505 (5)	C9—H9C	0.9600
C8—C7	1.510 (5)	C3—H3A	0.9300
			
O5—P1—O4	114.40 (15)	C4—C5—C6	116.7 (4)
O5—P1—O3	108.76 (16)	C4—C5—H5	121.7
O4—P1—O3	108.07 (15)	C6—C5—H5	121.7
O5—P1—O2	111.14 (13)	C3—C2—C1	116.2 (3)
O4—P1—O2	105.53 (15)	C3—C2—H2B	121.9
O3—P1—O2	108.77 (16)	C1—C2—H2B	121.9
P1—O3—H3	109.5	N2—C6—C5	132.6 (3)
P1—O2—H2	109.5	N2—C6—C1	106.3 (3)
C7—N2—C6	108.7 (3)	C5—C6—C1	121.1 (4)
C7—N2—H2A	125.6	N1—C7—N2	109.5 (3)
C6—N2—H2A	125.6	N1—C7—C8	124.1 (3)
C7—N1—C1	109.9 (3)	N2—C7—C8	126.3 (3)
C7—N1—H1	125.1	C5—C4—C3	121.8 (4)
C1—N1—H1	125.1	C5—C4—H4	119.1
O1—C8—C9	110.3 (3)	C3—C4—H4	119.1
O1—C8—C7	106.2 (3)	C8—C9—H9A	109.5
C9—C8—C7	113.2 (3)	C8—C9—H9B	109.5
O1—C8—H8	109.0	H9A—C9—H9B	109.5
C9—C8—H8	109.0	C8—C9—H9C	109.5
C7—C8—H8	109.0	H9A—C9—H9C	109.5
C8—O1—H1A	109.5	H9B—C9—H9C	109.5
C2—C1—N1	132.5 (3)	C2—C3—C4	122.3 (4)
C2—C1—C6	121.8 (4)	C2—C3—H3A	118.8
N1—C1—C6	105.7 (3)	C4—C3—H3A	118.8

### Hydrogen-bond geometry (Å, °)

**Table:**

*D*—H···*A*	*D*—H	H···*A*	*D*···*A*	*D*—H···*A*
O3—H3···O4^i^	0.82	1.76	2.532 (4)	156
O2—H2···O5^ii^	0.82	1.76	2.539 (3)	157
N2—H2A···O4	0.86	1.88	2.729 (4)	167
N1—H1···O5^iii^	0.86	1.81	2.659 (4)	167
O1—H1A···O2^iv^	0.82	2.23	2.971 (4)	150

Symmetry codes: (i) *x*−1, *y*, *z*; (ii) *x*−1/2, −*y*+1/2, −*z*+1; (iii) −*x*+3/2, −*y*+1, *z*−1/2; (iv) −*x*+2, *y*+1/2, −*z*+1/2.
